# Supplementary material for: Thinning Effect of Few-Layer Black Phosphorus Exposed to Dry Oxidation
Source: Nanomaterials (Basel). 2025 Jun 23;15(13):974. doi: 10.3390/nano15130974 (PMC12251546; doi:10.3390/nano15130974)
Supplement: Supplementary file 1 [file nanomaterials-15-00974-s001.zip › nanomaterials-3664292-supplementary.pdf]

Thinning Effect of Few-layer Black Phosphorus Exposed to Dry Oxidation

Qianyi Li [1,†], Hang Yang [3,†], Xiaofang Zheng [4,†], Yu Chen [1], Chuanxin Wang [1], Yujie Han [4], Yujing Guo [4], Xiaoming Zheng [1,]* and Yuehua Wei [2,]*

[1] Hunan Provincial Key Laboratory of Intelligent Sensors and Advanced Sensor Materials, School of Physics and Electronic Science, Hunan University of Science and Technology, Xiangtan 411201, China; 2208040428@mail.hnust.edu.cn (Q.L.); chen_yu@mail.hnust.edu.cn (Y.C.); 2408040305@mail.hnust.edu.cn (C.W.)

[2] School of Physics and Optoelectronics, Xiangtan University, Xiangtan 411105, China

[3] Beijing Blue Sky Innovation for Frontier Science, Beijing 100085, China; yanghang10@nudt.edu.cn

[4] Institute of Environmental Science, Shanxi University, Taiyuan 030006, China; 202212901005@email.sxu.edu.cn (X.Z.); yujiehan@sxu.edu.cn (Y.H.); guoyj@sxu.edu.cn (Y.G.)

* Correspondence: 1080098@hnust.edu.cn (X.Z.); yuehuawei@xtu.edu.cn (Y.W.)

† These authors contributed equally to this work.

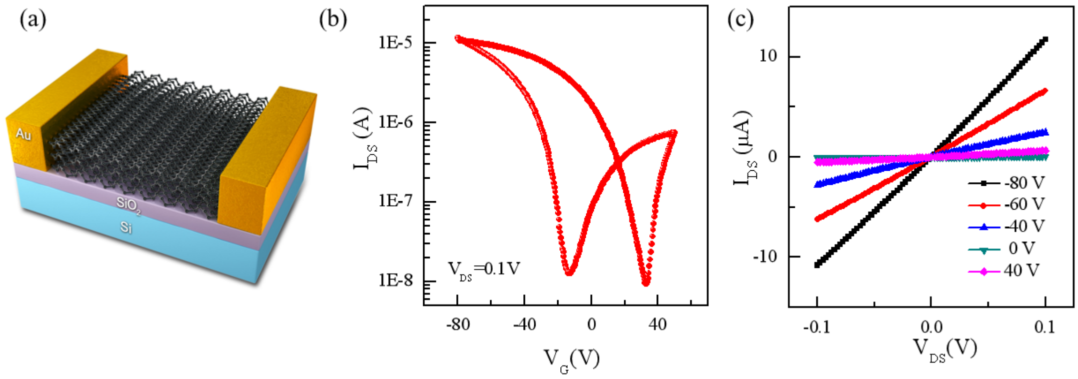

Figure S1 Characteristics of the few-layer BP. (a) Schematic diagram of the device structure. (b) Transfer characteristic of BP FET. (c) Output characteristic curve.

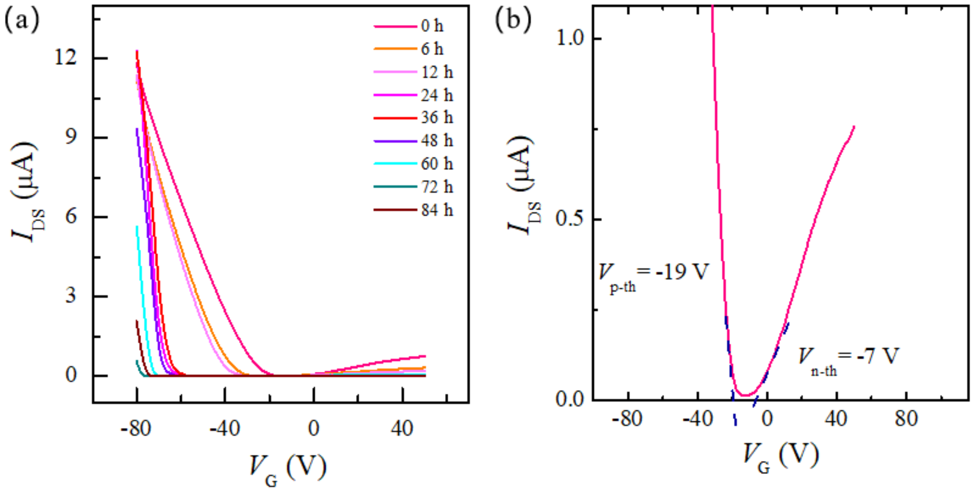

Figure S2. (a) Time dependent transfer characteristics (V_G from -80 V to 50 V) of BP FET in linear scale. (b) Magnified transfer characteristics of pristine BP FET (0 h)

close to the device off-state. The initial value of the ordinate is set to zero.

Following the previously reported methodology in bilayer MoS_2 FETs, the threshold voltages in the ambipolar transfer curves were utilized to extract the size of bandgap. At the threshold voltage of n-branch (labeled as $V_{n\text{-th}}$), the Fermi level at the source is aligned to the conduction band of BP; while the Fermi level at the drain moves to align with the valence band as V_G reaches the threshold voltage of p-branch (labeled as $V_{p\text{-th}}$). $V_{n\text{-th}}$ and $V_{p\text{-th}}$ are extracted in the linear plot rather than the logarithmic scale. Thus, time dependent transfer characteristics of BP FET in linear scale are shown in Figure S3a. The bandgap of BP can be estimated using the formula below:

$$E_g = e(V_{DS} + \frac{V_{n\text{-th}} - V_{p\text{-th}}}{\beta}) \qquad \text{(S1)}$$

Where β is the band movement factor:

$$\beta = 1 + C_T / C_{OX} \qquad \text{(S2)}$$

C_T and C_{OX} are the interface trap capacitance and oxide capacitance, respectively. Alternatively, close to the device off-state, SS equals $60\times\beta$ mV/decade, resulting in the extraction of β factor from experimentally measured SS in individual transfer plot. Here, to accurately extract the values of threshold voltage of two branches, the transfer characteristics need to be magnified close to the device off-state and the initial value of the ordinate should be set to zero. As demonstrated in Figure S3b, $V_{n\text{-th}}$ and $V_{p\text{-th}}$ are -7 V and -19 V for the pristine BP FET (0 h), respectively. Similarly, the same method can be used to extract the threshold voltages at other oxidation times (from 6 h to 84 h). It is to note that the calculated band-gap of pristine BP (0 h) is firstly normalized by theoretical value for bulk BP ~ 0.3 eV.

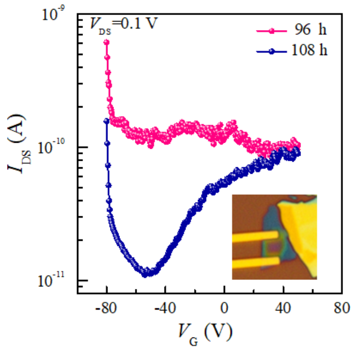

Figure S3. Transfer characteristics of BP FET in dry oxidation process after 96 h and 108 h.

As shown in Figure S3, after 96 h dry exposure, BP FET finally behaves no obvious transfer characteristics, indicating that the device broke down and could no longer work as a FET. Especially, the on-state current of BP FET after 108 h exposure only reaches 0.1 nA, which may be mainly come from the leakage current of gate dielectric layer. It is also evidenced by the optical characterization that the conductive channel has been completely oxidized.

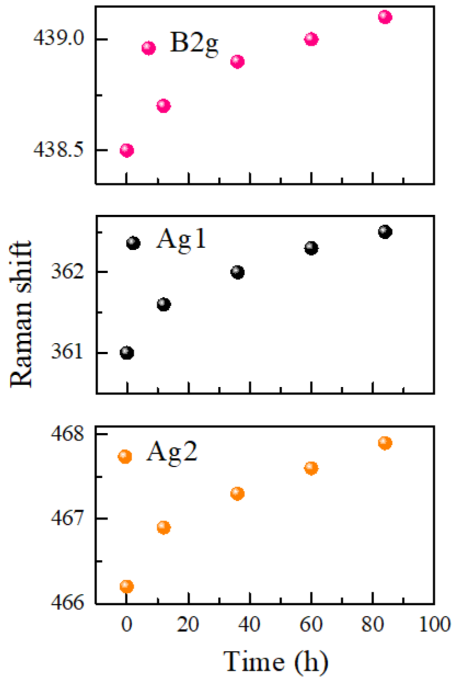

Figure S4. Layer-dependent Raman peak shift in phosphorene

To accurately compare the Raman peak intensities of BP flakes at different exposure time, the experimental conditions including the laser power and the laser focus position were kept the same through every experiment. Here, the laser power was measured by an optical power meter (Thorlabs PM121D) and the laser spot size was precisely adjusted to the minimum (focus) on sample surface. Additionally, the integration time and the accumulation numbers were all set as 1 s and 20 times, respectively. Finally, the SiO_2 signal in blank regions (Si substrate with no samples) was collected in every experiment to further normalize the Raman peak intensities.

The explanation regarding the variation in Si peak intensity at different time points in Figure S5a is provided below. This is due to that the transmittance of P_xO_y (rapidly formed after laser illumination, shown as Figure R3 and Figure R4) is much larger than BP (almost transparent in the visible range, *Phys. Rev. B* **2015**, 91, 85407), therefore leading to an increasing penetration of the laser light on Si substrate. At the end, the intensity of Si exhibits an obvious increase. This phenomenon is also in accordance with previous reports about Raman characterizations (λ = 532 nm) on a 5 nm-thick exfoliated BP sample on Si substrate (*Nat. Mater.* **2015**, 14, 826).

The mechanism is described as follows: Under the influence of covalent bonding, the P atoms collectively oscillate within the monolayer. When another layer of phosphorene is added and bonded by van der Waals forces, the oscillation of the P atoms is hindered. Hence, the corresponding Raman energy becomes smaller resulting in a red-shift. Here, the interlayer van der Waals force, which is dominant along the stacking direction in BP, plays an important role in the layer-dependent Raman characteristics. When the layer number increases, both the low-frequency rigid phosphorene layer breathing mode and shear mode red-shift and the correction on the interlayer van der Waals forces becomes weak. The layer-dependent Raman shifts can be effectively utilized to determine the thickness of phosphorene similar to graphene.

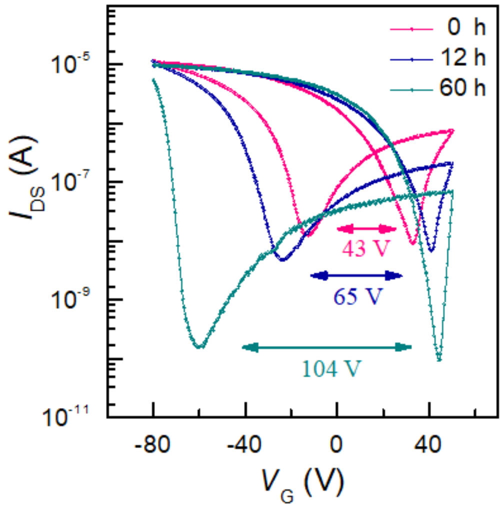

Figure S5 Transfer characteristic of BP FET.
